# Stiffness pulsation of the human brain detected by non-invasive time-harmonic elastography

**DOI:** 10.3389/fbioe.2023.1140734

**Published:** 2023-08-15

**Authors:** Tom Meyer, Bernhard Kreft, Judith Bergs, Erik Antes, Matthias S. Anders, Brunhilde Wellge, Jürgen Braun, Marvin Doyley, Heiko Tzschätzsch, Ingolf Sack

**Affiliations:** ^1^ Department of Radiology, Charité—University Medicine Berlin, Berlin, Germany; ^2^ Institute of Medical Informatics, Charité—University Medicine Berlin, Berlin, Germany; ^3^ Hajim School of Engineering and Applied Sciences, University of Rochester, Rochester, NY, United States

**Keywords:** ultrasound time-harmonic elastography, *in vivo* brain stiffness, brain pulsation, cerebrovascular compliance, intracranial pressure

## Abstract

**Introduction:** Cerebral pulsation is a vital aspect of cerebral hemodynamics. Changes in arterial pressure in response to cardiac pulsation cause cerebral pulsation, which is related to cerebrovascular compliance and cerebral blood perfusion. Cerebrovascular compliance and blood perfusion influence the mechanical properties of the brain, causing pulsation-induced changes in cerebral stiffness. However, there is currently no imaging technique available that can directly quantify the pulsation of brain stiffness in real time.

**Methods:** Therefore, we developed non-invasive ultrasound time-harmonic elastography (THE) technique for the real-time detection of brain stiffness pulsation. We used state-of-the-art plane-wave imaging for interleaved acquisitions of shear waves at a frequency of 60 Hz to measure stiffness and color flow imaging to measure cerebral blood flow within the middle cerebral artery. In the second experiment, we used cost-effective lineby-line B-mode imaging to measure the same mechanical parameters without flow imaging to facilitate future translation to the clinic.

**Results:** In 10 healthy volunteers, stiffness increased during the passage of the arterial pulse wave from 4.8% ± 1.8% in the temporal parenchyma to 11% ± 5% in the basal cisterns and 13% ± 9% in the brain stem. Brain stiffness peaked in synchrony with cerebral blood flow at approximately 180 ± 30 ms after the cardiac R-wave. Line-by-line THE provided the same stiffness values with similar time resolution as high-end plane-wave THE, demonstrating the robustness of brain stiffness pulsation as an imaging marker.

**Discussion:** Overall, this study sets the background and provides reference values for time-resolved THE in the human brain as a cost-efficient and easy-touse mechanical biomarker associated with cerebrovascular compliance.

## 1 Introduction

The brain is a pulsatile organ that expands by approximately half a milliliter due to the arterial pulse wave (Adams et al., 2019). This implies that cerebral vessel walls compliantly expand during the passage of the arterial pulse wave and transmit vascular stroke energy to the surrounding tissue (Wagshul et al., 2011). The transfer of mechanical energy from cerebral vessels to brain tissue decreases the propagation velocity of the pressure wave throughout the vascular tree and contributes to a more continuous perfusion profile within cerebral capillaries. Conversely, decreased absorption of pulsation energy due to aging or pathologic changes in the vessel walls can produce long-term damage to vessel walls and impair the transport of oxygen and nutrients. Probably for these reasons, high-pulse wave velocity and decreased vascular compliance have been associated with a greater risk of dementia (Wolters et al., 2017; Rouch et al., 2018).

Direct measurement of vessel compliance is challenging because it requires knowledge about the mechanical stress associated with a measured change in the blood flow (Zamir et al., 2018). Transcranial Doppler ultrasound or tailored MRI techniques (Yan et al., 2016; Wolters et al., 2017; Zwanenburg and van Osch, 2017) can quantify cerebral blood flow (Robba et al., 2018); however, mechanical stresses exerted by the pulse wave onto vessel walls and surrounding tissue are difficult to detect.

Here, we propose an alternative approach to study brain pulsatility and vascular compliance based on elastography. Elastography uses conventional imaging modalities to measure macroscopic tissue stiffness, for example, by sensing externally induced shear waves in soft tissues non-invasively (Ormachea and Parker, 2020). In vascularized tissue, such as the brain, the acquired stiffness maps reflect the mechanical properties of the effective medium, which change with perfusion, microfluid flow, and pressure (Poul et al., 2020). Our hypothesis is that cerebral arterial pulsation (CAP) induces measurable stiffness changes in the properties of the effective medium of the *in vivo* human brain.

Elastography of the brain has been mainly based on MRI (Hiscox et al., 2016; Yin et al., 2018; Nanjappa and Kolipaka, 2021; Herthum et al., 2022; Sack, 2022), while a few studies employed ultrasound (Ertl et al., 2018; Tzschatzsch et al., 2018) or optical methods (Ge et al., 2022). Unlike ultrasound elastography, magnetic resonance elastography (MRE) does not encounter acoustic shading through the bones of the skull and can produce high-resolution maps of the stiffness of human brain tissue (Manduca et al., 2021). On the downside, MRE is expensive, limited in availability, and requires acquisition times on the order of minutes, which are too long to detect CAP-induced stiffness changes without cardiac gating (Schrank et al., 2019). Ultrasound-based elastography is cost-effective, widely available, and has short acquisition times, but has limited ability to generate and encode shear waves in the brain (Ferraioli et al., 2015; Shiina et al., 2015; Cosgrove et al., 2017).

For the aforementioned reason, cerebral time-harmonic elastography (THE) has been developed based on sonography through the temples and acquisition of externally induced harmonic shear waves from 27 to 56 Hz, similar to the frequency range of MRE (Tzschatzsch et al., 2018). THE was shown to be sensitive to physiological alterations in cerebral blood flow due to the Valsalva maneuver (Tzschatzsch et al., 2018), hypercapnia (Kreft et al., 2020a), or dehydration (Kreft et al., 2020b), indicating the high sensitivity of brain stiffness to vascular compliance and intracranial pressure. In patients with intracranial hypertension, cerebral THE could detect stiffness changes before and after lumbar puncture (Kreft et al., 2022). However, owing to insufficient time resolution, none of these studies explored CAP-induced stiffness changes as a potential biomarker of cerebrovascular compliance.

Therefore, here, we developed time-resolved cerebral THE with a frame rate of 100 Hz to continuously capture the effects of CAP on brain stiffness over several heartbeats. In healthy volunteers, we performed two experiments. In one experiment, we used a high-end, plane-wave ultrasound scanner, which allowed us to acquire color flow images and stiffness maps in an interleaved fashion. This experiment was designed to ensure that established flow measurements and novel elastography captured the same physiological event on a shared time axis. In a second experiment, we used conventional line-by-line imaging to acquire only time-resolved stiffness changes without flow measurements, which facilitates clinical translation. Altogether, we aim to 1) report, for the first time, pulsation-induced changes in brain stiffness in the temporal lobe, basal cistern, and brain stem directly compared with cerebral blood flow and 2) demonstrate the implementation of this method on a clinical and cost-efficient ultrasound system based on conventional line-by-line imaging.

## 2 Materials and methods

### 2.1 Volunteers

The study was approved by the institutional review board of Charité—Universitätsmedizin Berlin (EA1/004/19). Written informed consent was obtained from all volunteers before participating in this study. Two groups of 10 healthy volunteers each (group 1: 3 women, age range: 21–52 years; group 2: 4 women, age range: 25–52 years) were prospectively included. Descriptive data of all volunteers (sex, age, BMI, heart rate, and mean arterial pressure) are summarized in Tables 1, 2. Inclusion criterion was no history of psychiatric disorders or head injury.

**TABLE 1:** Characteristics of the volunteers and individual CBF, global SWS, and peak delay time values detected by CBF-THE.

Volunteers					CBF				SWS				Delay time	
Sex	Age	BMI	HR	MAP	Mean	Systole	Diastole	Rel.	Mean	Systole	Diastole	Rel.	τ_R_	τ_Δ_
M/F	years	kg/m^2^	bpm	mmHg	cm/s	cm/s	cm/s	%	m/s	m/s	m/s	%	ms	ms
M	37	21.4	56	92	61	87	38	126	1.56	1.61	1.51	6.6	246	107
M	31	22.6	70	103	41	66	18	262	1.89	1.93	1.83	5.5	170	−9
M	27	21.0	73	84	87	101	71	42	1.93	2.01	1.86	8.1	215	9
M	21	22.9	86	85	79	101	45	126	1.92	2.00	1.85	8.1	162	−21
M	52	21.7	56	92	58	78	39	101	1.67	1.76	1.58	11.4	172	75
F	28	23.8	89	82	52	76	31	148	1.69	1.73	1.66	4.2	122	0
M	37	27.8	67	93	40	61	22	182	1.85	1.91	1.80	6.1	160	−9
M	28	23.5	70	90	40	57	21	172	1.86	1.95	1.78	9.6	189	−9
F	27	23.6	66	88	55	77	30	159	2.11	2.18	2.03	7.4	216	18
F	42	20.8	64	83	22	50	17	187	1.98	2.09	1.93	8.3	171	0
Mean	33	22.9	70	89	53	75	33	150	1.85	1.92	1.78	7.5	182	16
SD	9	1.9	10	6	18	17	15	55	0.15	0.16	0.15	2.0	34	39

**TABLE 2:** Characteristics and SWS values of volunteers acquired by c-THE.

Volunteers					SWS				
Sex	Age	BMI	HR	MAP	Mean	Systole	Diastole	Rel.	τ_Δ_
M/F	years	kg/m^2^	bmp	mmHg	m/s	m/s	m/s	%	ms
F	30	21	78	86	1.80	1.89	1.74	8.5	170
M	31	30	52	88	1.79	1.90	1.70	12.0	270
M	25	23	59	74	1.70	1.77	1.65	7.5	230
M	31	23	64	105	1.77	1.86	1.71	8.9	210
M	52	22	55	92	1.92	2.05	1.80	13.9	155
F	42	21	78	87	1.88	1.96	1.82	7.6	190
F	30	32	72	99	1.67	1.73	1.62	7.1	255
M	29	20	48	96	1.78	1.89	1.72	9.6	235
M	29	24	59	82	1.68	1.77	1.63	8.2	195
F	27	24	70	83	1.77	1.94	1.68	15.5	200
Mean	32	24	62	89	1.78	1.88	1.71	9.9	211
SD	8	4	11	8	0.08	0.10	0.07	2.9	36

### 2.2 Study protocol

In the first experiment, high-end, plane-wave imaging (Vantage 64LE, Verasonics, Washington, United States) for interleaved acquisitions of cerebral blood flow (CBF) using color flow imaging and shear wave speed (SWS) by THE was applied to group 1 (CBF-THE). In the second experiment, cost-efficient, line-by-line imaging (GAMPT mbH, Merseburg, Germany) was applied to the second group of volunteers for stiffness mapping (c-THE). In both CBF-THE and c-THE experiments, ECG data were continuously recorded for assigning CBF and SWS to cardiac phases. Each measurement took 8 s and was repeated five times to increase the statistical power.

### 2.3 Data acquisition

**FIGURE 1:**
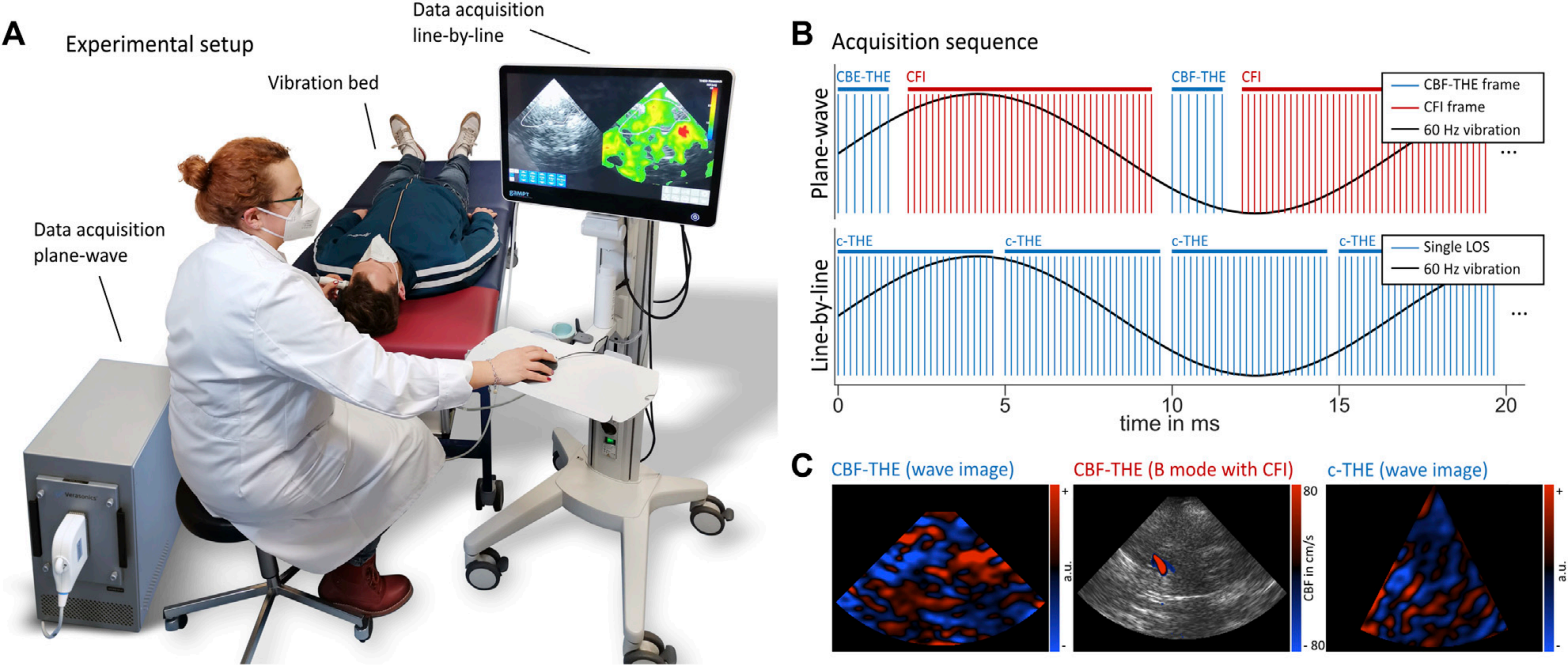
**(A)** Experimental setup for time-harmonic elastography (THE) with a vibration bed (GAMPT mbH, Merseburg, Germany), high-end, plane-wave ultrasound scanner (Vantage 64LE, Verasonics, Washington, United States) and cost-efficient, line-by-line THE device (GAMPT mbH, Merseburg, Germany). Volunteers were positioned in a supine position with the vibration unit underneath the back of their head. Measurements were performed through the temporal bone window, similar to clinical transcranial Doppler ultrasound. **(B)** Sequence diagram in the top shows the interleaved acquisition of THE and color flow imaging and in the bottom shows the line-by-line acquisition scheme. One image for plane-wave THE was composed of seven plane-wave angles, while a color flow image was composed of 40 frames. c-THE required 26 lines of sight (LOSs). **(C)** Representative wave images for both CBF-THE and c-THE, as well as the representative B-mode with CFI for CBF-THE.

Volunteers were investigated in a supine position on a custom-designed vibration bed (GAMPT mbH, Merseburg, Germany) with the head placed above the vibration unit for efficient transfer of harmonic vibration energy into the brain (Figure 1A) (Kreft et al., 2020a; Kreft et al., 2020b; Kreft et al., 2022). To avoid limitations in time resolution due to the decomposition of multifrequency wave setups, we used only single-frequency harmonic stimulations at 60 Hz, in contrast to previous THE studies. A high-end Vantage 64LE scanner (Verasonics, Washington, United States) equipped with a phased array transducer (P4-2v) with a center frequency of 3 MHz was used for CBF-THE. CBF (in cm/s) and SWS (in m/s) were acquired in an interleaved fashion (Figure 1B) with an effective frame rate of 100 Hz and up to a depth of 10 cm. The transducer was positioned at the temples, and an image plane in the temporal lobe of the brain was selected using color flow imaging of the middle cerebral artery overlaid to the anatomical B-mode (Figure 1C). For the elastography measurement, plane-wave compounding was used (seven plane-wave angles, −5° to 5°, with a pulse repetition frequency of 4 kHz). For color flow imaging, a burst of 40 frames was used (pulse repetition frequency of 5.5 kHz). For c-THE measurement, elastography data were acquired using a line-by-line ultrasound scanner (GAMPT mbH, Merseburg, Germany), as shown in Figure 1A, which was equipped with a phased-array transducer (P5-1S15-A6) having a center frequency of 3 MHz. The selected field-of-view was in the same region as the field-of-view in CBF-THE, with the same depth of up to 10 cm but a higher frame rate of 200 Hz and 26 lines of sight (Figure 1B), resulting in a slightly decreased insonation sector (Figure 1C). For this study, the entire data processing was newly developed to allow time-resolved SWS analysis.

### 2.4 Data processing

THE processing relied on the phase-difference extraction of the local axial displacement between adjacent in-phase and quadrature (IQ) frames. The temporal Fourier transform was then applied to the computed displacement data, and time-resolved complex wavefields (Figure 1C) were obtained using the filtered Hilbert transform with a bandpass filter centered at 60 Hz (Figure 1C). Due to undersampling, the vibration signal of 60 Hz acquired at a frame rate of 100 Hz is wrapped to the 40-Hz signal in the spectrum. The use of this wrapped signal instead of the original frequency is referred to as “controlled aliasing,” as proposed in our previous work (Tzschatzsch et al., 2016). The basic temporal resolution of the complex wavefields is related to both the frame rate of the full image acquisition (100 Hz) and the bandwidth of the Hilbert transformation. The bandpass filter employed in the Hilbert transform was Gaussian shaped with a width of 10 Hz and, thus, represents an empirically found tradeoff between noise robustness and high temporal resolution.

As the final step, time-resolved SWS maps were generated using wave-number based *k*-MDEV inversion (Cuiwei et al., 1995; Tzschatzsch et al., 2016). The inversion assumes plane shear waves $u\left(r,t\right)$ with complex wave number $k^{*}$ ( $k^{*}=k^{\prime }+ik^{\prime \prime }$ ), angular frequency *ω,* and amplitude *u*_*0*_ as solutions of the following wave equation:

$$u\left(r,t\right)=u_{0}∙e^{i\left(k^{*}∙r-\omega t\right)}.$$ (1)

*k*-MDEV first decomposed the complex-valued wavefields $u\left(r,t\right)$ obtained from the Hilbert transformation into eight complex wavefields ${\tilde{u}}_{d}$ for each propagation direction (*d*). For this, Gaussian-based spatiotemporal filters with a low-pass threshold of 555 rad/m for noise suppression and first-order derivative for suppression of compression waves were applied. The wave numbers were deduced from the phase gradient of $u_{d}$ :

$$k_{d}^{\prime }=\left\|\nabla \arg\left(u_{d}\right)\right\|,$$ (2a)

$$k_{d}^{\prime \prime }=\left\|\frac{\nabla \left|u_{d}\right|}{\left|u_{d}\right|}\right\|.$$ (2b)

The final maps of SWS were obtained by averaging over directions:

$$SWS=\frac{\omega \sum_{d}w}{\sum_{d}k_{d}^{\prime }w},with\;w={\left|u_{d}\right|}^{2}.$$ (3a)

Processing took approximately 15 min for the CBF-THE data and 30 s for the c-THE data. Regions of interest (ROIs) for the temporal lobe, basal cistern, and brain stem were manually drawn based on the anatomical B-mode image. SWS variations over time were analyzed after averaging within these regions for each time step. For color flow imaging processing, a region covering the middle cerebral artery was automatically selected through thresholding. A threshold of 5 cm/s was empirically set based on the amplitude of the background noise. A temporal moving boxcar filter of 3-pixel width was applied for noise suppression. The envelope of the flow spectrum was computed to obtain the time variation in CBF within the middle cerebral artery. R–R intervals were extracted from the acquired ECG signals with wavelet-based peak detection (Cuiwei et al., 1995) using the Haar wavelet (Sunkaria et al., 2010). For each volunteer, a normalized time axis covering one R–R interval was generated onto which the average of all THE and CBF signals acquired over 8 s and five repetitions was mapped. The resulting time curve of each volunteer and ROI were used to deduce the peak and minimum CBF and SWS values corresponding to cerebral peak systole and end-diastole, respectively. To quantify the pulsation, the relative difference between peak-systolic and end-diastolic SWS and CBF referenced to the end-diastolic value was computed. For comparing with the literature, the mean CBF and SWS values were calculated. Furthermore, the time delay of the SWS peak relative to the R-wave (τ_R_) and relative to the CBF peak (τ_Δ_) was quantified and tabulated.

### 2.5 Statistics

Relative changes in SWS and CBF over the R–R-interval were tested for each subregion using the paired Wilcoxon signed rank test. Delay times τ_R_ and τ_Δ_ were tested against zero using the Wilcoxon signed rank test. Pearson’s correlation coefficient was used to analyze possible correlations between the relative change in SWS and delay times τ_R_ and τ_Δ_ in the global ROI, with demographic parameters, such as age, BMI, heart rate, and mean arterial pressure, and the relative change in CBF. The relative change in SWS, mean SWS, and delay time τ_R_ obtained from CBF-THE was compared to c-THE using the Wilcoxon signed rank test. Nonparametric tests were chosen due to the small number of volunteers. All *p* values <0.05 were considered significant. The group size was estimated based on a power analysis assuming an effect size of 10%–20% at a 5% significance level and a power of 0.8 for the variability of stiffness parameters encountered in healthy adult subjects.

## 3 Results

**FIGURE 2:**
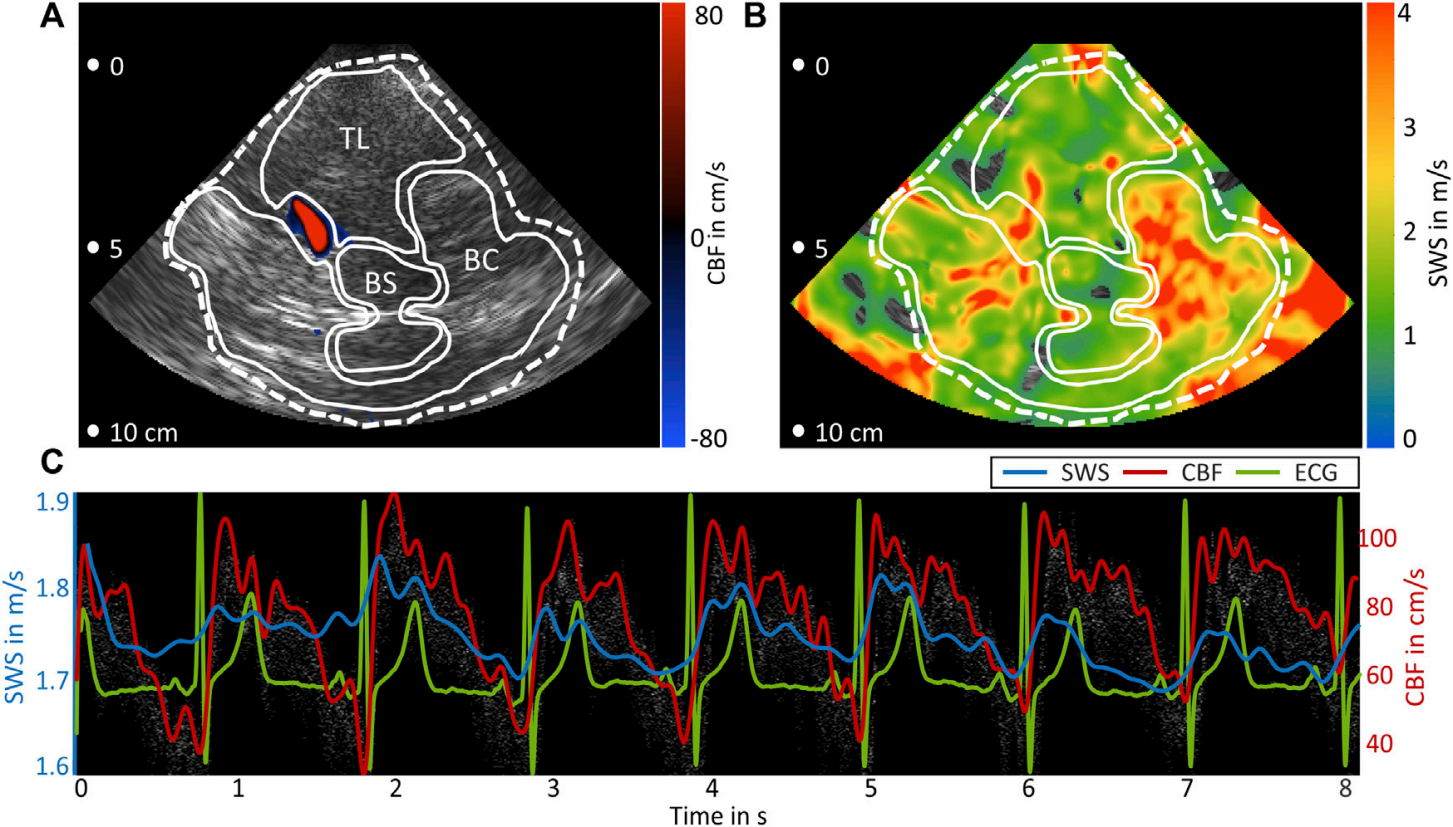
Representative data of B-mode, color flow imaging, and THE. **(A)** B-mode image overlaid with color flow of the middle cerebral artery (blue-to-red color scale). **(B)** SWS map. In **(A)** and **(B)**, the regions of interest are demarcated by white lines: temporal lobe parenchyma (TL), basal cistern (BC), and brain stem (BS) global region (dashed line). **(C)** Time curves of SWS (blue) and CBF (gray spectrum and red envelope) together with ECG (green).

In all volunteers, time-resolved SWS and, in the CBF-THE group, time-resolved CBF were obtained. Figure 2 shows the representative CBF and SWS data obtained by CBF-THE. The regions of interest were demarcated based on the anatomical landmarks in the B-mode image. Additionally, the time variations in SWS and CBF averaged within the global ROI are shown over a measure time of 8 s. The periodic change in both parameters in synchrony to the ECG suggests a relationship between brain stiffness and arterial blood flow in the temporal lobe.

**FIGURE 3:**
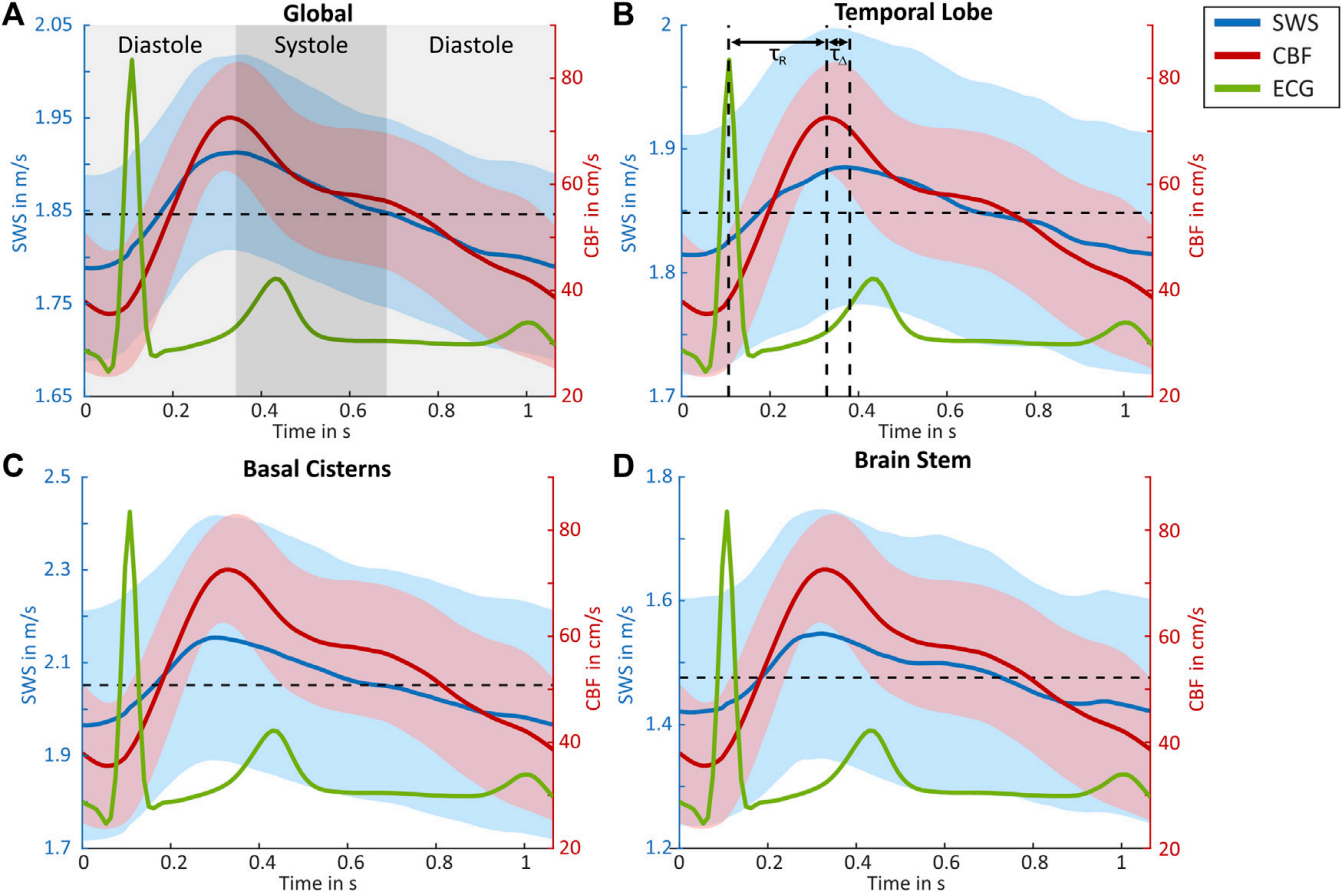
Group-averaged CBF and SWS values over normalized cardiac cycles. Shear wave speed (SWS, blue) with 95% confidence interval (blue area), cerebral blood flow (CBF, red) with the 95% confidence interval (red area), as well as ECG (green) for **(A)** global brain, **(B)** temporal lobe, **(C)** basal cisterns, and **(D)** brain stem regions. Cranial arterial systole and diastole are demarcated in **(A)**. In **(B)**, the peak systolic SWS and end diastolic SWS are demarcated by vertical dashed lines. τ_R_ indicates the time delay between ECG and SWS, while τ_Δ_ is the time delay between the SWS and Doppler peak. Horizontal dashed lines refer to the mean SWS values.

Group SWS and CBF values on a time axis normalized to one R–R-interval are presented for all four regions in Figure 3. As in Figure 2, both parameters vary synchronously over the cardiac phase, with higher relative changes and less group variation according to confidence intervals in CBF than those in SWS. The mean SWS was 1.85 ± 0.15 m/s in the global ROI, 1.85 ± 0.16 m/s in the temporal parenchyma, 2.05 ± 0.39 m/s in the basal cisterns, and 1.48 ± 0.29 m/s in the brain stem, whereas the mean CBF (54 ± 19 cm/s) was in the range of literature values (Krakauskaite et al., 2018), and SWS was slightly higher than that in previous cerebral THE studies due to the higher excitation frequency (Kreft et al., 2020b).

**FIGURE 4:**
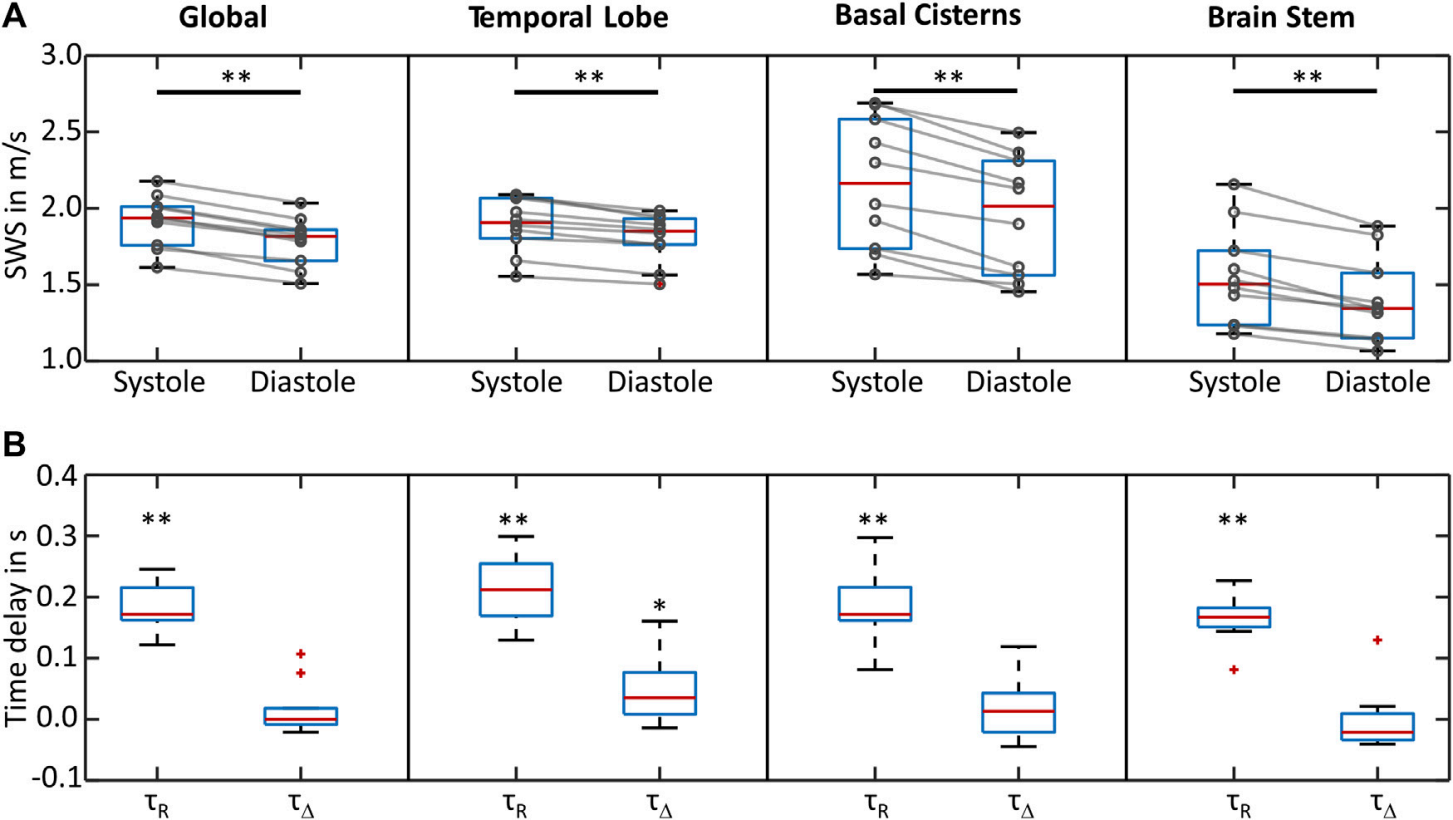
Group statistical plots of SWS and delay times. **(A)** SWS in peak systole and end diastole for all four regions of interest. In all regions, SWS decreased from systole to diastole. **(B)** Time delays between systolic ECG R-wave and peak SWS (τ_R_), as well as those between the peak SWS and peak CBF (τ_Δ_) for all examined regions of interest. **p < 0.01 and *p < 0.05.

In all regions, a significant relative decrease from peak systolic to end diastolic SWS values was observed (Figure 4A), with 7.5% ± 2.0% in the global ROI, 4.8% ± 1.8% in the temporal parenchyma, 11% ± 5% in the basal cisterns, and 13% ± 9% in the brain stem. For CBF, a corresponding relative decrease of 139% ± 59% was observed, as shown in Table 1. Time delays between the R peak and peak systolic SWS (τ_R_) were 180 ± 30 ms in the global ROI, 210 ± 50 ms in the temporal parenchyma, 180 ± 60 ms in the basal cisterns, and 160 ± 40 ms in the brain stem. In all regions except the temporal lobe, there was no significant time delay between the peak systolic SWS and peak systolic CBF (τ_Δ_). In the temporal lobe, τ_Δ_ of 50 ± 50 ms was observed, suggesting a slight delay in the SWS wave after the CBF wave (Figure 4B). No significant correlations were observed between the mean SWS and physiological parameters such as age, sex, and BMI. Moreover, no correlation was found between the relative SWS changes and blood pressure and delay times and heart rate. All SWS and CBF values and delay times acquired by CBF-THE are summarized in Table 1.

Group SWS within the global ROI measured by c-THE was similar to CBF-THE, with a mean SWS of 1.78 ± 0.08 m/s, relative SWS changes of 9.9% ± 2.9%, and a delay in the SWS peak to the R peak of 211 ± 36 ms. There was no significant difference in the values obtained by CBF THE (mean SWS: *p* = 0.21, relative SWS changes: *p* = 0.06, τ_R_: *p* = 0.13). Data acquired by c-THE are summarized in Table 2.

## 4 Discussion

To the best of our knowledge, this is the first study in which changes in brain stiffness due to cerebral arterial pulsation have been directly observed without cardiac gating and time averaging, that is, quasi-real time. We observed a change in SWS in synchrony to CBF pulsation, from which we concluded that CAP significantly affected brain stiffness. This effect was present in all regions, which underscores the relevance of the relative SWS change as a potential biomarker of cerebrovascular compliance. It should be noted that THE was less constrained by accurately locating anatomic landmarks than color flow imaging, which is limited to the middle cerebral artery. As a result, the overall time required, including probe positioning, was shorter for THE than for color flow imaging. Because there was no significant temporal shift between the SWS peak and the CBF peak of arterial pulsation, we can conclude that SWS measurement provides a robust and cost-effective imaging marker, that is, complementary to Doppler imaging.

Given the extensive literature on elastography in highly perfused organs, we speculate that the observed variation in brain stiffness is related to cerebrovascular compliance (Hirsch et al., 2012; Hirsch et al., 2014a; Hirsch et al., 2014b; Parker, 2014; Parker, 2015; Arani et al., 2018; Marticorena Garcia et al., 2018; Forouhandehpour et al., 2021; Solamen et al., 2021). Specifically, brain stiffness has been shown by MRE and THE to increase with cerebral artery dilation due to hypercapnia (Hetzer et al., 2019; Kreft et al., 2020a) or the Valsalva maneuver (Tzschatzsch et al., 2018; Herthum et al., 2021). As shown by MRE, cerebral stiffness of deep gray matter correlates with perfusion pressure rather than cerebral blood flow alone (Hetzer et al., 2018). This means that the vessel walls dilate during the passage of the pulse wave, causing a global stiffening of the tissue in which the vessels are embedded. In simple terms, when vascular pressure increases in encapsulated (non-expandible) organs such as the brain or liver, whether by increasing the total blood volume due to water uptake (Ipek-Ugay et al., 2016; Kreft et al., 2020b) or by transient passage of the arterial pulse wave as observed in this study, the tissue stiffens. Conversely, tissue softening has been reported in the spleen, which may expand with increased blood volume, likely due to the increased volume of fluid in the tissue with no change in pressure (Hirsch et al., 2014a; Meyer et al., 2022).

An exception here is our own previous work in which cardiac-gated MRE was used to measure CAP-induced stiffness changes (Schrank et al., 2019). In this study, a series of stiffness maps were generated, each composed of signals from 81 heartbeats, which, in combination with finger pulse triggering, led to uncertainties in the timing of stiffness peaks relative to CAP. The partially different results of this previous study compared with those of the current study emphasize the relevance of the simultaneous measurement of blood flow and stiffness—as currently only possible by THE in a plane-wave setup. Nevertheless, the question remains unresolved as to why THE is so sensitive to intracranial pressure, such as in patients before and after lumbar puncture (Kreft et al., 2022), whereas mixed results have been reported based on MRE (Kolipaka et al., 2018).

Further studies investigating the effects of different states of perfusion and pressure on the temporal modulation of cerebral stiffness are required to better understand the observed stiffness variation. In this context, the effects of dehydration and water uptake on brain stiffness have been studied based on cerebral THE (Kreft et al., 2020b). However, these studies were limited to time-averaged measurements, as opposed to the time-resolved measurements made possible through this study. Additionally, it would be interesting to see if an altered cerebral blood flow in patients with vascular male formations, vascular dementia, or intracranial hypertension changes the observed pulsation patterns (Lauriola et al., 2018; Liu et al., 2020; Kreft et al., 2022).

The main limitation to our study is the lack of clinical data. It would be interesting to see how stiffness pulsation changes upon the relief of intracranial hypertension in patients undergoing lumbar puncture. A possible change in stiffness pulsation over a wider age range and in conditions such as mild cognitive impairment and dementia is also warranted for future studies. In addition, the relatively small number of subjects precludes a conclusive analysis of physiological effects on changes in brain stiffness, as well as the assessment of possible timing differences between CBF and SWS waves. The insignificant correlation of τ_R_ with the heart rate may be due to the fact that we only investigated healthy volunteers with a relatively narrow range of heart rates. In future studies including patients with tachy- or bradycardia, we expect τ_R_ to be sensitive to the heart rate. However, this study focused on method development and first-time demonstration of CAP-induced stiffness alterations to provide a reference for larger cohort studies in a clinical setting.

## 5 Conclusion

In summary, our study demonstrated the feasibility of time-resolved cerebral THE for the assessment of cerebrovascular compliance based on pulsations of tissue stiffness. Plane-wave imaging-based THE showed that brain stiffness in healthy volunteers changes from minimum values during cerebral diastole to maximum values during cerebral systole in the order of 10% with variation across anatomical regions. This direct relationship between the stiffness wave and arterial blood pulse wave could make THE a sensitive imaging marker for brain compliance changes due to cerebrovascular pulsation, which could be used for diagnostics and treatment monitoring. Furthermore, relative stiffness changes measured by line-by-line-based THE was shown to be a cost-effective alternative to plane-wave-based THE that is ready to use in the clinic for the noninvasive measurement of brain stiffness associated with intracranial pressure and cerebrovascular compliance.

## Data Availability

The raw data supporting the conclusion of this article will be made available by the authors, without undue reservation.

## References

[B1] Adams A. L. Kuijf H. J. Viergever M. A. Luijten P. R. Zwanenburg J. J. M. (2019). Quantifying cardiac-induced brain tissue expansion using DENSE. NMR Biomed. 32 (2), e4050. 10.1002/nbm.4050 30575151 PMC6519010

[B2] Arani A. Min H. K. Fattahi N. Wetjen N. M. Trzasko J. D. Manduca A. (2018). Acute pressure changes in the brain are correlated with MR elastography stiffness measurements: initial feasibility in an *in vivo* large animal model. Magn. Reson Med. 79 (2), 1043–1051. 10.1002/mrm.26738 28488326 PMC5811891

[B3] Cosgrove D. Barr R. Bojunga J. Cantisani V. Chammas M. C. Dighe M. (2017). WFUMB guidelines and recommendations on the clinical use of ultrasound elastography: part 4. Thyroid. Ultrasound Med. Biol. 43 (1), 4–26. 10.1016/j.ultrasmedbio.2016.06.022 27570210

[B4] Cuiwei L. Chongxun Z. Changfeng T. (1995). Detection of ECG characteristic points using wavelet transforms. IEEE Trans. Biomed. Eng. 42 (1), 21–28. 10.1109/10.362922 7851927

[B5] Ertl M. Raasch N. Hammel G. Harter K. Lang C. (2018). Transtemporal investigation of brain parenchyma elasticity using 2-D shear wave elastography: definition of age-matched normal values. Ultrasound Med. Biol. 44 (1), 78–84. 10.1016/j.ultrasmedbio.2017.08.1885 28982629

[B6] Ferraioli G. Filice C. Castera L. Choi B. I. Sporea I. Wilson S. R. (2015). WFUMB guidelines and recommendations for clinical use of ultrasound elastography: part 3: liver. Ultrasound Med. Biol. 41 (5), 1161–1179. 10.1016/j.ultrasmedbio.2015.03.007 25800942

[B7] Forouhandehpour R. Bernier M. Gilbert G. Butler R. Whittingstall K. Van Houten E. (2021). Cerebral stiffness changes during visual stimulation: differential physiological mechanisms characterized by opposing mechanical effects. Neuroimage Rep. 1 (2), 100014. 10.1016/j.ynirp.2021.100014 PMC12172913 40567862

[B8] Ge G. R. Song W. Nedergaard M. Rolland J. P. Parker K. J. (2022). Theory of sleep/wake cycles affecting brain elastography. Phys. Med. Biol. 67 (22), 225013. 10.1088/1361-6560/ac9e40 PMC9999375 36317278

[B9] Herthum H. Hetzer S. Kreft B. Tzschätzsch H. Shahryari M. Meyer T. (2022). Cerebral tomoelastography based on multifrequency MR elastography in two and three dimensions. Front. Bioeng. Biotechnol. 10, 1056131. 10.3389/fbioe.2022.1056131 36532573 PMC9755504

[B10] Herthum H. Shahryari M. Tzschatzsch H. Schrank F. Warmuth C. Gorner S. (2021). Real-time multifrequency MR elastography of the human brain reveals rapid changes in viscoelasticity in response to the Valsalva maneuver. Front. Bioeng. Biotechnol. 9 (335), 666456. 10.3389/fbioe.2021.666456 34026743 PMC8131519

[B11] Hetzer S. Birr P. Fehlner A. Hirsch S. Dittmann F. Barnhill E. (2018). Perfusion alters stiffness of deep gray matter. J. Cereb. Blood Flow. Metab. 38 (1), 116–125. 10.1177/0271678x17691530 28151092 PMC5757437

[B12] Hetzer S. Dittmann F. Bormann K. Hirsch S. Lipp A. Wang D. J. (2019). Hypercapnia increases brain viscoelasticity. J. Cereb. Blood Flow. Metab. 39 (12), 2445–2455. 10.1177/0271678x18799241 30182788 PMC6893988

[B13] Hirsch S. Guo J. Reiter R. Papazoglou S. Kroencke T. Braun J. (2014a). MR elastography of the liver and the spleen using a piezoelectric driver, single-shot wave-field acquisition, and multifrequency dual parameter reconstruction. Magn. Reson Med. 71 (1), 267–277. 10.1002/mrm.24674 23413115

[B14] Hirsch S. Guo J. Reiter R. Schott E. Buning C. Somasundaram R. (2014b). Towards compression-sensitive magnetic resonance elastography of the liver: sensitivity of harmonic volumetric strain to portal hypertension. J. Magn. Reson Imaging 39 (2), 298–306. 10.1002/jmri.24165 23649541

[B15] Hirsch S. Klatt D. Freimann F. Scheel M. Braun J. Sack I. (2012). *In vivo* measurement of volumetric strain in the human brain induced by arterial pulsation and harmonic waves. Magn. Reson Med. 70 (3), 671–683. 10.1002/mrm.24499 23008140

[B16] Hiscox L. V. Johnson C. L. Barnhill E. McGarry M. D. Huston J. van Beek E. J. (2016). Magnetic resonance elastography (MRE) of the human brain: technique, findings and clinical applications. Phys. Med. Biol. 61 (24), R401–R437. 10.1088/0031-9155/61/24/r401 27845941

[B17] Ipek-Ugay S. Tzschatzsch H. Hudert C. Marticorena Garcia S. R. Fischer T. Braun J. (2016). Time harmonic elastography reveals sensitivity of liver stiffness to water ingestion. Ultrasound Med. Biol. 42 (6), 1289–1294. 10.1016/j.ultrasmedbio.2015.12.026 26971462

[B18] Kolipaka A. Wassenaar P. A. Cha S. Marashdeh W. M. Mo X. Kalra P. (2018). Magnetic resonance elastography to estimate brain stiffness: measurement reproducibility and its estimate in pseudotumor cerebri patients. Clin. Imaging 51, 114–122. 10.1016/j.clinimag.2018.02.005 29459315 PMC6087505

[B19] Krakauskaite S. Thibeault C. LaVangie J. Scheidt M. Martinez L. Seth-Hunter D. (2018). Normative ranges of transcranial Doppler metrics. Acta Neurochir. Suppl. 126, 269–273. 10.1007/978-3-319-65798-1_53 29492573

[B20] Kreft B. Bergs J. Shahryari M. Danyel L. A. Hetzer S. Braun J. (2020b). Cerebral ultrasound time-harmonic elastography reveals softening of the human brain due to dehydration. Front. Physiol. 11, 616984. 10.3389/fphys.2020.616984 33505319 PMC7830390

[B21] Kreft B. Tzschatzsch H. Schrank F. Bergs J. Streitberger K. J. Waldchen S. (2020a). Time-resolved response of cerebral stiffness to hypercapnia in humans. Ultrasound Med. Biol. 46 (4), 936–943. 10.1016/j.ultrasmedbio.2019.12.019 32001088

[B22] Kreft B. Tzschatzsch H. Shahryari M. Haffner P. Braun J. Sack I. (2022). Noninvasive detection of intracranial hypertension by novel ultrasound time-harmonic elastography. Invest. Radiol. 57 (2), 77–84. 10.1097/rli.0000000000000817 34380993

[B23] Lauriola M. Mangiacotti A. D'Onofrio G. Cascavilla L. Paris F. Paroni G. (2018). Neurocognitive disorders and dehydration in older patients: clinical experience supports the hydromolecular hypothesis of dementia. Nutrients 10 (5), 562. 10.3390/nu10050562 29751506 PMC5986442

[B24] Liu H. Wang D. Leng X. Zheng D. Chen F. Wong L. K. S. (2020). State-of-the-Art computational models of circle of willis with physiological applications: A review. IEEE Access 8, 156261–156273. 10.1109/access.2020.3007737

[B25] Manduca A. Bayly P. J. Ehman R. L. Kolipaka A. Royston T. J. Sack I. (2021). MR elastography: principles, guidelines, and terminology. Magn. Reson Med. 85 (5), 2377–2390. 10.1002/mrm.28627 33296103 PMC8495610

[B26] Marticorena Garcia S. R. Grossmann M. Lang S. T. Tzschatzsch H. Dittmann F. Hamm B. (2018). Tomoelastography of the native kidney: regional variation and physiological effects on *in vivo* renal stiffness. Magn. Reson Med. 79 (4), 2126–2134. 10.1002/mrm.26892 28856718

[B27] Meyer T. Tzschatzsch H. Wellge B. Sack I. Kroncke T. Martl A. (2022). Valsalva maneuver decreases liver and spleen stiffness measured by time-harmonic ultrasound elastography. Front. Bioeng. Biotechnol. 10, 886363. 10.3389/fbioe.2022.886363 35711644 PMC9195299

[B28] Nanjappa M. Kolipaka A. (2021). Magnetic resonance elastography of the brain. Magnetic Reson. Imaging Clin. N. Am. 29 (4), 617–630. 10.1016/j.mric.2021.06.011 34717849

[B29] Ormachea J. Parker K. J. (2020). Elastography imaging: the 30 year perspective. Phys. Med. Biol. 65 (24), 1361–6560. 10.1088/1361-6560/abca00 33181486

[B30] Parker K. J. (2014). A microchannel flow model for soft tissue elasticity. Phys. Med. Biol. 59 (15), 4443–4457. 10.1088/0031-9155/59/15/4443 25049224

[B31] Parker K. J. (2015). Experimental evaluations of the microchannel flow model. Phys. Med. Biol. 60 (11), 4227–4242. 10.1088/0031-9155/60/11/4227 25973729

[B32] Poul S. S. Ormachea J. Hollenbach S. J. Parker K. J. (2020). Validations of the microchannel flow model for characterizing vascularized tissues. Fluids 5 (4), 228. 10.3390/fluids5040228 34707336 PMC8547714

[B33] Robba C. Cardim D. Sekhon M. Budohoski K. Czosnyka M. (2018). Transcranial Doppler: a stethoscope for the brain-neurocritical care use. J. Neurosci. Res. 96 (4), 720–730. 10.1002/jnr.24148 28880397

[B34] Rouch L. Cestac P. Sallerin B. Andrieu S. Bailly H. Beunardeau M. (2018). Pulse wave velocity is associated with greater risk of dementia in mild cognitive impairment patients. Hypertension 72 (5), 1109–1116. 10.1161/hypertensionaha.118.11443 30354804

[B35] Sack I. (2022). Magnetic resonance elastography from fundamental soft-tissue mechanics to diagnostic imaging. Nat. Rev. Phys. 5, 25–42. 10.1038/s42254-022-00543-2

[B36] Schrank F. Warmuth C. Tzschatzsch H. Kreft B. Hirsch S. Braun J. (2019). Cardiac-gated steady-state multifrequency magnetic resonance elastography of the brain: effect of cerebral arterial pulsation on brain viscoelasticity. J. Cereb. Blood Flow. Metab. 40, 991–1001. 10.1177/0271678x19850936 31142226 PMC7181097

[B37] Shiina T. Nightingale K. R. Palmeri M. L. Hall T. J. Bamber J. C. Barr R. G. (2015). WFUMB guidelines and recommendations for clinical use of ultrasound elastography: part 1: basic principles and terminology. Ultrasound Med. Biol. 41 (5), 1126–1147. 10.1016/j.ultrasmedbio.2015.03.009 25805059

[B38] Solamen L. M. McGarry M. D. J. Fried J. Weaver J. B. Lollis S. S. Paulsen K. D. (2021). Poroelastic mechanical properties of the brain tissue of normal pressure hydrocephalus patients during lumbar drain treatment using intrinsic actuation MR elastography. Acad. Radiol. 28 (4), 457–466. 10.1016/j.acra.2020.03.009 32331966 PMC7575616

[B39] Sunkaria R. K. Saxena S. C. Kumar V. Singhal A. M. (2010). Wavelet based R-peak detection for heart rate variability studies. J. Med. Eng. Technol. 34 (2), 108–115. 10.3109/03091900903281215 20059305

[B40] Tzschatzsch H. Kreft B. Schrank F. Bergs J. Braun J. Sack I. (2018). *In vivo* time-harmonic ultrasound elastography of the human brain detects acute cerebral stiffness changes induced by intracranial pressure variations. Sci. Rep. 8 (1), 17888. 10.1038/s41598-018-36191-9 30559367 PMC6297160

[B41] Tzschatzsch H. Nguyen Trong M. Scheuermann T. Ipek-Ugay S. Fischer T. Schultz M. (2016). Two-dimensional time-harmonic elastography of the human liver and spleen. Ultrasound Med. Biol. 42 (11), 2562–2571. 10.1016/j.ultrasmedbio.2016.07.004 27567061

[B42] Wagshul M. Eide P. Madsen J. (2011). The pulsating brain: A review of experimental and clinical studies of intracranial pulsatility. Fluids Barriers CNS 8, 5. 10.1186/2045-8118-8-5 21349153 PMC3042979

[B43] Wolters F. J. Zonneveld H. I. Hofman A. van der Lugt A. Koudstaal P. J. Vernooij M. W. (2017). Cerebral perfusion and the risk of dementia A population-based study. Circulation 136 (8), 719–728. 10.1161/circulationaha.117.027448 28588075

[B44] Yan L. Liu C. Y. Smith R. X. Jog M. Langham M. Krasileva K. (2016). Assessing intracranial vascular compliance using dynamic arterial spin labeling. Neuroimage 124, 433–441. 10.1016/j.neuroimage.2015.09.008 26364865 PMC4651759

[B45] Yin Z. Romano A. J. Manduca A. Ehman R. L. Huston J. 3rd (2018). Stiffness and beyond: what MR elastography can tell us about brain structure and function under physiologic and pathologic conditions. Top. Magn. Reson Imaging 27 (5), 305–318. 10.1097/rmr.0000000000000178 30289827 PMC6176744

[B46] Zamir M. Moir M. E. Klassen S. A. Balestrini C. S. Shoemaker J. K. (2018). Cerebrovascular compliance within the rigid confines of the skull. Front. Physiology 9, 940. 10.3389/fphys.2018.00940 PMC6056744 30065667

[B47] Zwanenburg J. J. M. van Osch M. J. P. (2017). Targeting cerebral small vessel disease with MRI. Stroke 48 (11), 3175–3182. 10.1161/strokeaha.117.016996 28970280

